# Silencing lncRNA ZFAS1 or elevated microRNA-135a represses proliferation, migration, invasion and resistance to apoptosis of osteosarcoma cells

**DOI:** 10.1186/s12935-019-1049-x

**Published:** 2019-12-03

**Authors:** Zilong Zhao, Xiafei Lin, Yunhui Tong, Wenxia Li

**Affiliations:** 1 Upper Limb Injury Dept 2, Luo Yang Orthopedic-Traumatological Hospital, Luoyang, 476000 Henan China; 2 Upper Limb Injury Dept 1, Luo Yang Orthopedic-Traumatological Hospital, Luoyang, 476000 Henan China; 3 Department of Hip Injuries, Luo Yang Orthopedic-Traumatological Hospital, Luoyang, 476000 Henan China; 4 Emergency Department, Luo Yang Orthopedic-Traumatological Hospital, NO. 82 Qiming Nan Road, Luoyang, 476000 Henan China

**Keywords:** Osteosarcoma, Long noncoding RNA Zinc finger antisense 1, MicroRNA-135a, Proliferation, Migration, Invasion, Apoptosis

## Abstract

**Background:**

Osteosarcoma (OS) is still a disease with high mortality from malignant tumors in children and adolescents. Due to its poor treatment, this study explored the involvement of lncRNA ZFAS1/microRNA-135a (miR-135a)/apurinic/apyrimidinic exonuclease 1 (APEX1) axis in the regulation of OS growth and metastasis.

**Methods:**

ZFAS1, miR-135a and APEX1 expression in OS tissues and cells were tested by RT-qPCR and western blot analysis. MG63 cells were transfected with sh-ZFAS1, miR-135a mimic or their controls to unearth theirs functions in the proliferation, colony formation, migration, invasion, cycle entry and apoptosis of MG63 cells by MTT and EdU, colony formation assays, flow cytometry, and Transwell assay, severally. The proliferation related factor (Ki-67, CyclinD1), apoptosis related factor (Bax, Bcl-2) and migration related factor (MMP2, MMP9) protein levels were tested. Tumor volume and weight were detected by subcutaneous tumor xenograft in nude mice.

**Results:**

Overexpressed ZFAS1 and APEX1, and down-regulated miR-135a existed in OS tissues and cells. Silenced ZFAS1 or elevated miR-135a inhibited colony formation and proliferation, cycle progression, migration and invasion while promoted apoptosis of MG63 cells. Silenced ZFAS1 or elevated miR-135a suppressed tumor volume and weight of OS in vivo. LncRNA ZFAS1 promoted APEX1 expression by competitively binding with miR-135a.

**Conclusion:**

This study indicates that silenced ZFAS1 or up-regulated miR-135a restrained migration, proliferation and invasion and promoted apoptosis of OS MG63 cells. This study provides a possible theoretical basis for studying the regulatory mechanism of ZFAS1/miR-135a/APEX1 signaling axis on the growth and metastasis of OS.

## Background

Osteosarcoma (OS), a most general cancer, results in plenty of deaths and has various kinds of classifications [1]. The obvious symptom includes onset of pain and swelling in the influenced bone in OS [2]. OS is an infrequent tumor of mesenchymal origin, mostly often influencing adolescents and young adults [3]. At present, radiotherapy, surgery, neoadjuvant and adjuvant chemotherapy are the main therapies for OS. Although there has been great improvement in improving clinical results, the prognosis of OS patients is still poor due to pulmonary metastasis and tumor recurrence after resection [4]. Due to the poor prognosis of OS, it is urgent to seek new therapeutic targets to improve the prognosis of this disease.

The long noncoding RNA Zinc finger antisense 1 (lncRNA ZFAS1), a new lncRNA located at 20q13 and is expressed in lots of tissues and organs [5]. ZFAS1 plays an oncogenic part in some kinds of cancers [6]. Liu et al. have found that up-regulated ZFAS1 in OS samples and cell lines is apparently connected with poor prognosis of OS patients [7]. Another study has demonstrated that ZFAS1 with other elements axis might play an significant part in OS development, which could be a possible biomarker for the diagnosis and therapy of OS [8]. In addition, microRNA-135a (miR-135a), a miR existing in the miR-135 superfamily, has been found to take part in the tumorigenesis of all kinds of cancers [9]. For example, miR-135a has been assessed to predict lymph node metastasis and tumor stage in gastric cancer and suppress the tumor growth in gastric cancer [10]. Several miRNAs have been verified to play a part in OS development. For instance, miR-193a exerts its function in the OS chemoresistance, therefore, it might be regarded as a useful biomarker for OS prognosis [11]. What’s more, miRNAs such as miR-214 and miR-138 are involved in OS cell growth, exosome function, tumor growth microenvironment and phenotype [12]. Apurinic/apyrimidinic exonuclease 1 (APEX1) is an essential enzyme in the base excision repair pathway whose function is the repair of DNA resulted from oxidative and alkylation damage containing single-strand breaks [13]. Recently, APEX1 has been regarded as a target for cancer therapeutics because its high expression have been found to be related to resistance to radiotherapy, chemotherapy and poor survival in a general spectrum of cancers [13]. A study offered evidence indicating that APEX1 is a key prognosis marker and a possible therapeutic target for OS [13]. This study was for the research of the involvement of lncRNA ZFAS1/miR-135a/APEX1 axis in regulating OS growth and metastasis.

## Materials and methods

### Ethics statement

The study was permitted by the Institutional Review Board of Luo Yang Orthopedic-Traumatological Hospital and followed the tenets of *the Declaration of Helsinki*. Written informed consent was received from participants. The experiment was approved by the Institutional Animal Care and Use Committee of Luo Yang Orthopedic-Traumatological Hospital. Animals were treated humanely using approved procedures in line with the principles of animal protection, animal welfare and ethics.

### Study subjects

From January 2015 to December 2018, patients pathologically diagnosed with OS in Luo Yang Orthopedic-Traumatological Hospital were chosen in the study. Patients were included if they met the following criteria: (1) specimens were with typical pathological morphology and confirmed with OS by clinical, radiological test, and biopsy; (2) there were complete medical records and preserved wax blocks before and after biopsy; (3) the specimens were not carried out with radiotherapy and chemotherapy before specimen collection; (4) the specimens were assessed by two experienced pathologists for pathological diagnosis; (5) the cases of specimens were only for operative treatment; (6) the detailed contact information and follow-up of specimens were available. Exclusion criteria: (1) clinical pathological data proved that before the collection of specimens, radiotherapy and chemotherapy was carried out in patients with OS or those who were not clear about radiotherapy and chemotherapy (implementation of the radiotherapy and chemotherapy after specimen collection were not excluded); (2) Pegat disease, benign lesions (such as bone infarction and fibrous dysplasia), dedifferentiated chondrosarcoma and other diseases existed in cases of specimens after OS.

The samples of tumor tissue from 34 cases of patients with OS were collected with 200 mg each, labeled and placed at − 70 °C for later use. Among them, there were 18 males of patients with OS and 16 cases of females; the age of patients was 29–57 years, in the mean of (42.4 ± 10.6) years. According to World Health Organization (WHO) classification, there were 14 cases of osteoblast type, 12 cases of chondroblast type, 8 cases of inoblast type. Another 30 cases of specimens of bone tissue (normal tissue) from patients with simple fractures in the same period were selected as the control group.

### Cell selection and culture

Human OS cells 143B, U2OS, Saos2, MG63 and human normal osteoblasts hFOB1.19 were purchased from Shanghai Oulu biotechnology Co., Ltd. (Shanghai, China). All kinds of cells were cultured in Dulbecco’s Modified Eagle Medium (DMEM) (Gibco, Grand Island, NY, USA) containing 10% fetal bovine serum (FBS) at 37 °C with 5% CO_2_. The cells were in passage culture when the cell confluence reached 80–90%. After 2 to 3 of stable passage, the cells in logarithmic growth phase were taken, and ZFAS1 and miR-135a and APEX1 mRNA expression in all cells were tested by reverse transcription quantitative polymerase chain reaction (RT-qPCR). The APEX1 protein expression was examined by western blot analysis. MG63 cells with the greatest differential expression of human normal osteoblast cell line hFOB1.19 were selected for subsequent experiments.

### Cell grouping and transfection

MG63 cells in the logarithmic growth phase (2 × 10^5^ cells/well) were seeded into a 6-well cell culture plate. When adhering to the wall and reaching 80% confluence, the cells started to be transfected in line with the instructions of lipofectamine™ 2000 kit (11668-027, Invitrogen, CA, USA). Each transfected sequence was diluted by 250 μL serum-free DMEM medium (Shanghai GenePharma Co., Ltd., Shanghai, China), the final concentration of cells was 50 nM). The other 250 μL serum-free DMEM medium was used for dilution of 5 μL lipofectamine™ 2000. The above two solution were mixed and incubated for 20 min and added to the cell culture well. The cells were incubated for 6 h at 37 °C with 5% CO_2_ and saturated humidity. The medium containing the transfection solution in the well was discarded, and replaced with DMEM medium including 10% FBS with continuous culture for following experiments. The MG63 cells were assigned into 7 groups, including the blank (no transfection treatment), the sh-negative control (NC) (transfected with silenced ZFAS1 NC), the sh-ZFAS1 (transfected with silenced ZFAS1 plasmid), the mimic-NC (transfection of miR-135a mimic NC), the miR-135a mimic (transfected with miR-135a mimic), the sh-ZFAS1 + inhibitor NC (transfected with sh-ZFAS1 + down-regulated miR-135a NC), the sh-ZFAS1 + miR-135a inhibitor (transfected with sh-ZFAS1 + down-regulated miR-135a) groups.

### 3-(4,5-dimethyl-2-thiazolyl)-2,5-diphenyl-2-H-tetrazolium bromide (MTT) assay

MG63 cells of all groups were seeded to a 96-well plate at a density of 3 × 10^4^ cells/mL, and cultured at 37 °C with 5% CO_2_ for 48 h. Each group was set with 8 parallel wells, and 20 μL of fresh MTT solution was joined to per well (concentration of 5 mg/mL). After 4-h reaction, the cells were added with 200 μL of Dimethyl Sulphoxide. After sufficient dissolution, the microplate reader was used to detect the optical density (OD) value of each group at 490 nm. Three times repeats of the experiment were existed and the results were recorded.

### 5-Ethynyl-2′-deoxyuridine (EdU) assay

The DNA replication ability of each group of MG63 cells was examined via using a Cell-light EdU luminescence assay kit (RiboBio, Guangzhou, China). Each group of cells was routinely detached, re-suspended and counted, and seeded into a 96-well plate at 1.0 × 10^4^ cells/well, with 3 parallel wells in each group. After incubating for 2 h with 100 μL of 50 μM EdU, the cells were fixed with 4% paraformaldehyde for 20 min and then further incubated with 2% glycine for 15 min. Then the liquid was dried, and 150 μL of permeabilizing agent was added for permeabilization. The cells were then treated according to the EdU kit instructions. Five fields of view were randomly taken and photographed under a fluorescence microscope (FSX100, Olympus, Tokyo, Japan). Blue fluorescence represented all cells, and red fluorescence was the replicated cells infiltrated by EdU. The percentage of EdU-positive cells was calculated.

### Colony formation assay

The colony formation rate of MG63 cells in each group was tested by colony formation assay. The cells in the logarithmic growth phase were detached with 0.25% trypsin, adjusted to a cell concentration of 1 × 10^5^ cells/mL, and further diluted to 1 × 10^3^ cells/mL. The cell suspension with 200 μL was seeded into a petri dish (200 cells per dish). The dish was gently rotated to disperse evenly, and the placed in an incubator for 14-day incubation. When the colonized spots could be seen by the naked eye, the culture was stopped and the supernatant was discarded. The cells were washed with phosphate buffered saline (PBS), and fixed with 5 mL of 4% methanol for 15 min. After the fixed solution was removed, cells were stained with Giemsa staining solution for 30 min, the staining solution was removed by rinsing with tap water. The plate was inverted and superimposed on a transparent grid of film, and then observed under a microscope, for calculating the colony formation rate of each group of cells. The cell colony formation rate was expressed as the ratio of the amount of colonies to seeded cells.

### Flow cytometry

The MG63 cells of each group were collected, and some cells were tested for cell cycle. Propidium Iodide (PI)-Rnase A of 100 μL was added and incubated for 15 min in darkness. The different DNA content in each period of each cell cycle was analyzed by flow cytometry. The cells were detached, centrifuged and collected, re-suspended with pre-cooled 75% ethanol, and fixed at − 20 °C overnight. The cells were re-suspended by 450 μL of PBS in per sample, joined with 100 μL Rnase A with water bath at 37 °C for 30 min, and stained with 400 μL of PI and equably mixed. After cells’ staining for 30 min at 4 °C in darkness, the cell cycle distribution was measured and analyzed by using flow cytometer (FACSCalibur, BD company, New Jersey, USA). The MG63 cells of all groups were collected. Each group was added with pre-cooled 100 μL of 1 × binding buffer to re-suspend the cells. Five μL of Annexin and 5 μL of PI were added in sequence, and kept away from light for 15 min. Apoptosis was examined by flow cytometer. The results were judged as follows: Annexin V was as the horizontal axis and PI as the vertical axis; The mechanical injury cell was in the upper left quadrant; and the late apoptotic cell or the necrotic cell, the upper right quadrant; the negative normal cell, the lower left quadrant; the early apoptotic cell, the lower right quadrant.

### Transwell assay

After 24-h transfection, the adjusted density of the detached cells was 1 × 10^5^ cells/mL. The cell suspension with 200 μL was seeded into the upper transwell chamber and serum-free medium was added. The lower chamber was joined with complete medium consisting of 10% serum, and cultured at 37 °C with 5% CO_2_. After 24 h, the chamber was removed, and the un-migrated cells in the upper chamber were removed with a cotton swab. The cells were fixed with formaldehyde at 37 °C for 30 min, and the fixed solution was removed. Then the cells were fixed with 0.5% crystal violet staining solution for 20 min. With the inverted microscope, five fields were randomly selected to count and photograph the migrated cells. The transwell chamber of the cell invasion assay was pre-coated with 50 μL of Matrigel solution, and the remaining steps were the same as that of cell migration test.

### Scratch test

The MG63 cells in the logarithmic growth phase were seeded into a 6-well cell culture plate at 1 × 10^5^ cells/mL, and cultured for 24 h. The marker was used to make a mark on the back of the cell culture plate, at intervals of 0.5 cm, and crossing through wells, at least 5 lines each well. Each group of culture medium was changed. The cells were treated with mitomycin for 1 h. The scratching existed by employing a ruler and a pipette head with a range of 20 μL. After cells’ washing with PBS, the delineated cells were removed and the medium (without serum) was added. The cells were incubated in an incubator with 5% CO_2_ at 37 °C, and the cell healing rate was sampled and checked at 0th h and 24th h, respectively. Five fields in each sample visual were randomly selected for measurement of distances. The migration rate of all groups was calculated by the formula: healing rate = 100% × (0th h scratch distance − 24th h scratch distance)/0th h scratch distance.

### RT-qPCR

Total RNA of tissues and cells was extracted by Trizol (Takara, Dalian, China) method. The concentration and purity of RNA were determined. The RNA was reversely transcribed into cDNA in line with the instructions of the reverse transcription kit (K1621, Fermentas, Maryland, NY, USA). ZFAS1, miR-135a, and APEX1 primer sequences were designed and submitted to Shanghai Genechem Co., Ltd. (Shanghai, China) for synthesis (Table 1). The mRNA expression of each gene were tested by a real-time PCR kit (Takara, Dalian, China). RT-qPCR (ABI 7500, ABI, Foster City, CA, USA) instrument was employed for the detection. With U6 as internal reference of miR-135a, glyceraldehyde phosphate dehydrogenase (GAPDH) for ZFAS1 and APEX1, the relative expression of each target gene was calculated by 2^−ΔΔCt^ method.

**Table 1:** Primer sequence

Gene	Primer sequence (5′–3′)
ZFAS1	Forward: 5′-ACGTGCAGACATCTACAACCT-3′
	Reverse: 5′-TACTTCCAACACCCGCAT-3′
miR-135a	Forward: 5′-CTGGTAGGTATGGCTTTTTATTC-3′
	Reverse: 5′-TCAACTGGTGTGGTGGAGTC-3′
APEX1	Forward: 5′-CTCACCCAGTGGCAAATCAG-3′
	Reverse: 5′-TGTCACACACTGCAGGCAA-3′
U6	Forward: 5′-CTCGCTTCGGCAGCACA-3′
	Reverse: 5′-AACGCTTCACGAATTTGCGT-3′
GAPDH	Forward: 5′-TCCCATCACCATCTTCCA-3′
	Reverse: 5′-CATCACGCCACAGTTTTCC-3′

*ZFAS1* zinc finger antisense 1, *miR-135a* microRNA-135a, *APEX1* apurinic/apyrimidinic exonuclease 1, *GAPDH* glyceraldehyde-3-phosphate dehydrogenase

### Western blot analysis

Total protein of cells and tissues were extracted. The protein concentration was determined in line with the instructions of bicinchoninic acid kit (Boster Biological Technology Co., Ltd., Wuhan, Hubei, China). The extracted protein was added to the loading buffer and then boiled at 95 °C for 10 min, and each well was loaded with 30 μg. Protein separation was carried out by 10% polyacrylamide gel (Boster Biological Technology Co., Ltd., Wuhan, Hubei, China) electrophoresis. The protein was transferred onto polyvinylidene difluoride membrane and the membrane was sealed with 5% bovine serum albumin for 1 h. The membrane was added with primary antibody Ki-67 (1:1000), APEX1 (1:5000), MMP9 (1:1000) (Abcam, Cambridge, UK), CyclinD1 (1:1000), Bax (1:1000), Bcl-2 (1:1000), MMP2 (1:500) (Santa Cruz Biotechnology, Santa Cruz, California, USA), and GAPDH (1:2000) (Jackson Immuno Research, Grove, Pennsylvania, USA). The membrane was incubated at 4 °C for 24 h to 48 h, then with horseradish peroxidase-labeled secondary antibody (1:500, Jackson Immuno Research, Grove, Pennsylvania, USA) for 1-h incubation. Images were obtained using Odyssey two-color infrared fluorescence scanning imaging system. The gray value of the band was measured using the Quantity One image analysis software. The differences between the groups were compared by the ratio of each target band to the internal reference band.

### Fluorescence in situ hybridization (FISH)

The subcellular localization of ZFAS1 was predicted by using the bioinformatics website (http://lncatlas.crg.eu/). The subcellular localization of lncRNA ZFAS1 in MG63 cells was then identified via using FISH technology. The experiment followed the instructions of Ribo™ lncRNA FISH Probe Mix (Red) (RiBoBio), and the specific method was as follows. The coverslip was placed in a 24-well culture plate. MG63 cells were seeded at 6 × 10^4^ cells/well for the cell confluence of about 80%. The slides were removed, and the cells were fixed with 1 mL of 4% paraformaldehyde. After treatment with proteinase K, glycine and acetamidine reagent, the cells were added with 250 μL of pre-hybrid solution, and incubated at 42 °C for 1 h. Then the pre-hybrid solution was removed, and the cells were added with 250 μL hybridization solution containing the probe of lncRNA ZFAS1 (300 ng/mL) and hybridized overnight at 42 °C. After 3 times-washing with Phosphate Buffered Saline plus Tween-20 (PBST), the 4′,6-diamidino-2-phenylindole solution (ab104139, 1: 100, Abcam, Shanghai, China) diluted with PBST was added to dye the nucleus. Lastly, the plate was sealed with an anti-fluorescent quenching agent, and observed under a fluorescence microscope (Olympus, Tokyo, Japan) and photographed.

### Dual luciferase reporter gene assay

The binding sites of lncRNA ZFAS1 and miR-135a were predicted and analyzed by the bioinformatics website (https://cm.jefferson.edu/rna22/Precomputed/). The binding relationship between ZFAS1 and miR-135a was verified by the dual luciferase reporter gene assay. The synthetic ZFAS1 3′untranslated regions (UTR) gene fragment was introduced into pMIR-reporter via employing the endonuclease sites Bamh1 and Ecor1 (Huayueyang Biotechnology Co., Ltd., Beijing, China). In the ZFAS1 wild type (WT), a complementary sequence mutation site of the seed sequence was designed. The target fragment was inserted into the pMIR-reporter plasmid by using T4 DNA ligase after restriction endonuclease digestion. The correctly sequenced luciferase reporter plasmids WT and mutant type (MUT) were co-transfected with mimic NC and miR-135a mimic into MG63 cells (Shanghai Beinuo Biotechnology Co., Ltd., Shanghai, China). After 48 h of transfection, the cells were harvested and lysed, and luciferase activity was measured via using a luciferase assay kit (BioVision, San Francisco, CA, USA) and Glomax 20/20 luminometer (Promega, Madison, Wisconsin, USA).

The targeting relationship between miR-135a and APEX13 and the binding site of miR-135a and APEX1 3′UTR were predicted via using bioinformatics software (http://www.targetscan.org/vert_72/). The APEX1 3′UTR promoter region sequence containing the miR-135a binding site was synthesized, and the APEX1 3′UTR WT plasmid (APEX1-WT) was constructed. Based on this plasmid, the APEX1 3′UTR mutant (MUT) plasmid (APEX1-MUT) was constructed by mutating the binding site. Next steps followed the procedure of the purchased plasmid extraction kit (Promega, Madison, USA). The cells in logarithmic growth phase was seeded into a 96-well plate. At a cell confluence of about 70%, Lipofectamine 2000 was used for transfection, and the APEX1-WT and APEX1-MUT plasmids were mixed with mimic NC and miR-135a mimic plasmid, respectively, and then co-transfected with MG63 cells. After 48-h transfection, the cells were harvested and lysed, and luciferase activity was detected by using a luciferase assay kit.

### RNA-pull down assay

Biotin-labeled miR-135a WT plasmid and miR-135a MUT plasmid (50 nM each) were transfected into cells, respectively. After 48-h transfection, the cells were harvested and incubated with specific cell lysates (Ambion, Austin, Texas, USA) for 10 min. The sample of lysate with 50 mL was then dispensed. Residual lysate was incubated with M-280 streptavidin magnetic beads (Sigma, St. Louis, MO, USA) pre-coated RNase-free and yeast tRNA (Sigma, St. Louis, MO, USA) for 3 h at 4 °C. Then the cells were washed twice with cold lysate, three times with low salt buffer, and once with high salt buffer. An antagonistic miR-135a probe was established as a NC. Total RNA was extracted with Trizol, and ZFAS1 expression was detected by RT-qPCR.

### Tumor xenograft in nude mice

A sum of 35 BALB/c nude mice aged 4–5 weeks (Hunan SJA Laboratory Animal Co., Ltd., (Hunan, China) were raised under specific pathogen-free conditions, and adjusted to the new environment in the animal room for 1 week. The state of the animals was observed every day to ensure food and water supply (all used after strict disinfection). Thirty-five nude mice were divided into seven groups, including the blank, the sh-NC, the sh-ZFAS1, the mimic-NC, the miR-135a mimic, the sh-ZFAS1 + inhibitor NC, and the sh-ZFAS1 + miR-135a inhibitor groups, with 5 mice in each group. MG63 cells were collected after detachment with trypsin, washed with serum-free medium, and made into cell suspension. The cell suspension was absorbed under sterile conditions with 1 mL sterile syringe. Nude mice in each group were subcutaneously injected with 100 μL of single cell suspension (2 × 10^6^ cells in total) at a concentration of 2 × 10^7^ cells/mL in the back, respectively. Nude mice were continued to be raised under sterile conditions. The growth of subcutaneous tumors in nude mice was observed every day. The tumors length and width of the nude mice were measured every 3 days, and the tumor volume was calculated based on the measured quantitative values. The formula for calculating tumor volume was: (length × width)/2. The average subcutaneous tumor volume of each group of 5 nude mice was calculated, and the tumor growth curves of nude mice in each group were plotted. On the 21st day after the tumor was implanted, the nude mice were euthanized. The subcutaneous tumors of nude mice were completely exfoliated, and the tumor weights were weighed separately. The average tumor weight of each group of nude mice was calculated for statistical analysis.

### Statistical analysis

All data were statistically analyzed via using SPSS 21.0 (IBM-SPSS, Inc, Chicago, IL, USA) statistical software. Measurement data were expressed as mean ± standard deviation. The data subjected to normal distribution were compared between two groups via using independent sample t-test. One-way analysis of variance (ANOVA) was employed for comparisons among multiple groups, and Tukey’s multiple comparisons test was used for pairwise comparisons after ANOVA analysis. Pearson analysis was used for correlation analysis. *P* < 0.05 was regarded as statistical significance.

## Results

### Overexpressed ZFAS1, APEX1, and down-regulated miR-135a are found in OS tissues

**Fig. 1:**
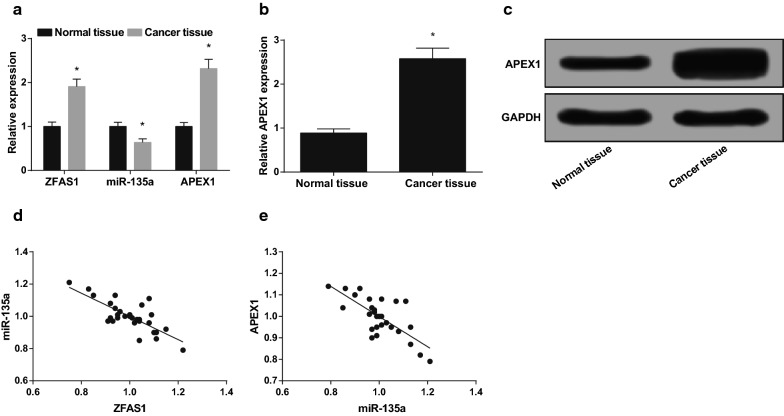
Elevated ZFAS1, APEX1, and declined miR-135a arefound in OS tissues. **a** Levels of ZFAS1, miR-135a, and APEX1 mRNA in OS tissues and normal tissues detected by RT-qPCR. **b** The protein band and expression of APEX1 in OS tissues and normal tissues. **c** The correlation analysis between ZFAS1 and miR-135a. **d** The correlation analysis between miR-135a and APEX1. Data in the figure were measurement data, in the form of mean ± standard deviation. * vs the normal tissue, *P *< 0.05

The levels of ZFAS1, miR-135a and APEX1 mRNA and the protein level of APEX1 in normal tissue and OS tissue were detected by RT-qPCR and western blot analysis. There were elevated ZFAS1 and APEX1 and declined miR-135a in OS tissue in contrast with normal tissues (all *P* < 0.05) (Fig. 1a, b). In OS tissues, the correlation of the expression between ZFAS1 and miR-135a, along with miR-135a and APEX1 was detected by Pearson correlation analysis. The results found that the expression of ZFAS1 with miR-135a has a negative correlation (r = − 0.768, *P* < 0.05); miR-135a expression was negatively correlated with APEX1 expression (r = − 0.735, *P* < 0.05) (Fig. 1c, d).

### Highest expression of ZFAS1, APEX1 and lowest expression of miR-135a are found in MG63 cells

**Fig. 2:**
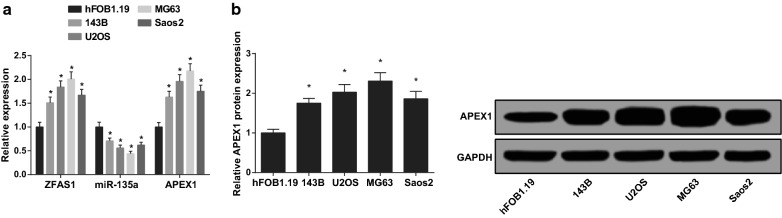
Highest ZFAS1, APEX1 and lowest miR-135a are found in MG63 cells. **a** The levels of ZFAS1, miR-135a, and APEX1 mRNA in each cell line tested by RT-qPCR. **b** The protein band and expression of APEX1 in each cell line. The data in the figure were all measurement data, in the form of mean ± standard deviation. * vs hFOB1.19 cells, *P* < 0.05

The levels of ZFAS1, miR-135a and APEX1 mRNA in hFOB1.19, 143B, U2OS, Saos2 and MG63 cells were detected by RT-qPCR. It was found that there was increased ZFAS1 and APEX1 and decreased miR-135a in 143B, U2OS, Saos2 and MG63 cells by contrast with hFOB1.19 cells (all *P* < 0.05). There were highest ZFAS1 and APEX1 and lowest miR-135a in MG63 cells, which had the largest difference in comparison with hFOB1.19 cells (Fig. 2a). The protein levels of APEX1 in 143B, U2OS, Saos2 and MG63 cells were tested by western blot analysis. In contrast to hFOB1.19 cells, the protein levels of APEX1 in 143B, U2OS, Saos2 and MG63 were elevated (all *P *< 0.05). MG63 cells had the highest APEX1 with the greatest difference from hFOB1.19 cells (Fig. 2b). In summary, MG63 cells were selected for subsequent experiments.

### Silenced ZFAS1 or up-regulated miR-135a inhibits colony formation ability and proliferation of MG63 cells

**Fig. 3:**
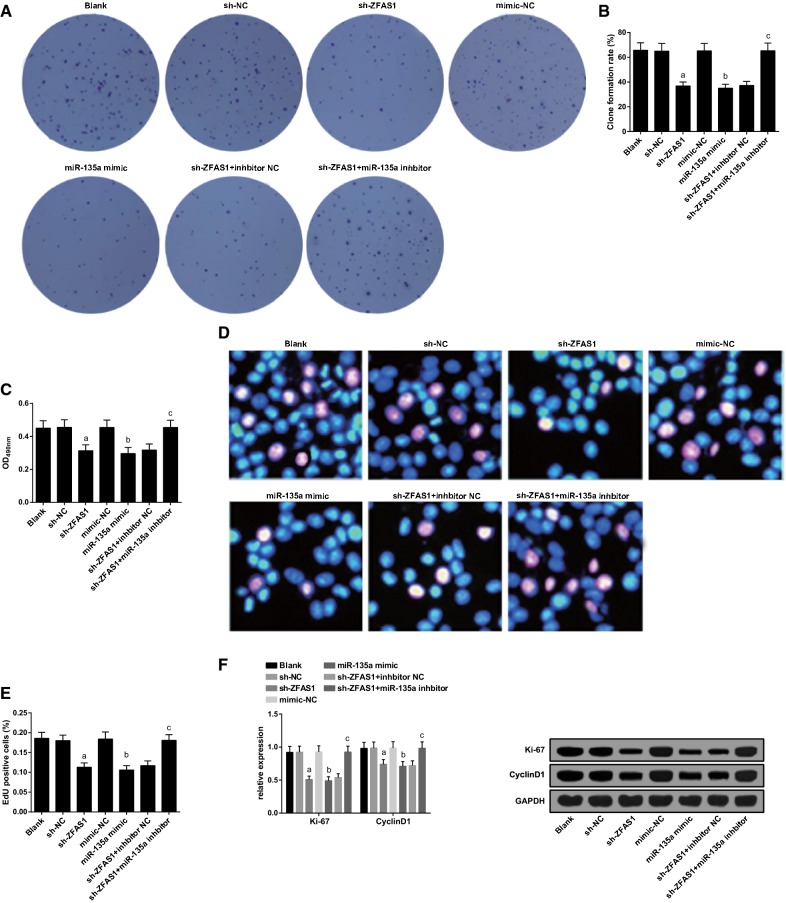
Down-regulated ZFAS1 or up-regulated miR-135a restrains colony formation ability and proliferation of MG63 cells. **A** The colony formation ability of MG63 cells in each group. **B** Quantification results in **A**. **C** MG63 cell proliferation tested by MTT assay. **D** EdU assay for detecting proliferation of MG63 cells. **E** Quantification results in **D**. **F** Ki-67 and CyclinD1 protein band and expression in each group of MG63 cells. The data in the figure were all measurement data, in the form of mean ± standard deviation; a vs the sh-NC group, *P* < 0.05; b vs the mimic-NC group, *P* < 0.05; c vs the sh-ZFAS1 + inhibitor NC group, *P* < 0.05

The colony formation ability, proliferation, proliferation-related protein Ki-67 and CyclinD1 expression of MG63 cells in each group were detected by MTT and EdU assays and western blot analysis severally. Versus the blank group, there was no apparent changes in the cell colony formation ability, the cell proliferation rate, Ki-67 and CyclinD1 expression of cells in the sh-NC and the mimic NC groups (all *P* > 0.05). By contrast with the sh-NC group and the mimic-NC group, there were declined cell colony formation ability, cell proliferation rate, Ki-67 and CyclinD1 expression in the sh-ZFAS1 group and the miR-135a mimic group (all *P* < 0.05). Relative to the sh-ZFAS1 + inhibitor NC group, there were elevated cell colony formation ability, cell proliferation rate, Ki-67 and CyclinD1 expression in the sh-ZFAS1 + miR-135a inhibitor group (all *P* < 0.05) (Fig. 3A–F).

### Declined ZFAS1 or elevated miR-135a restrains MG63 cell cycle progression and promotes apoptosis

**Fig. 4:**
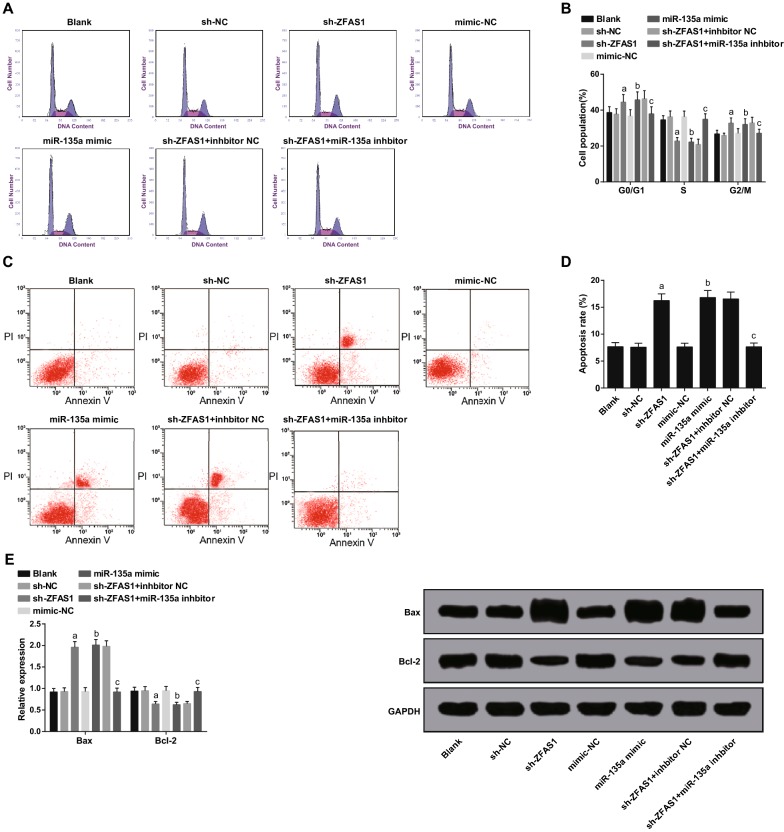
Declined ZFAS1 or elevated miR-135a represses MG63 cell cycle entry and facilitates apoptosis. **A** The cell cycle distribution of MG63 cells in each group detected by flow cytometry. **B** Quantification results in **A**. **C** Apoptosis of MG63 cells in each group tested by flow cytometry. **D** Quantification results in **C**. **E** Bax and Bcl-2 protein bands and expression in each group of MG63 cells. The data in the figure were all measured data, in the form of mean ± standard deviation; a vs the sh-NC group, *P* < 0.05; b vs the mimic-NC group, *P* < 0.05; c vs the sh-ZFAS1 + inhibitor NC group, *P* < 0.05

The cell cycle, apoptosis, apoptosis-related protein Bax and Bcl-2 expression of MG63 cells was tested by flow cytometry and western blot analysis severally. Versus the blank group, no apparent changes occurred in the proportion of cells in G0/G1, S and G2/M phases, the apoptosis rate as well as Bax and Bcl-2 expression of MG63 cells in the sh-NC and the mimic-NC groups (all *P* > 0.05). There were elevated apoptotic rate, proportion of cells in G0/G1 and G2/M phases, Bax protein expression, while declined proportion of cells in S phase and Bcl-2 protein expression in the sh-ZFAS1 and the miR-135a mimic groups in contrast with the sh-NC and the mimic NC groups severally (all *P* < 0.05). In contrast to the sh-ZFAS1 + inhibitor NC group, there were declined apoptotic rate, proportion of cells in G0/G1 and G2/M phases, Bax protein expression, and elevated proportion of cells in S phase and Bcl-2 protein expression in the sh-ZFAS1 + miR-135a inhibitor group (all *P* < 0.05) (Fig. 4A–E).

### Silenced ZFAS1 or up-regulated miR-135a inhibits MG63 cell migration and invasion

**Fig. 5:**
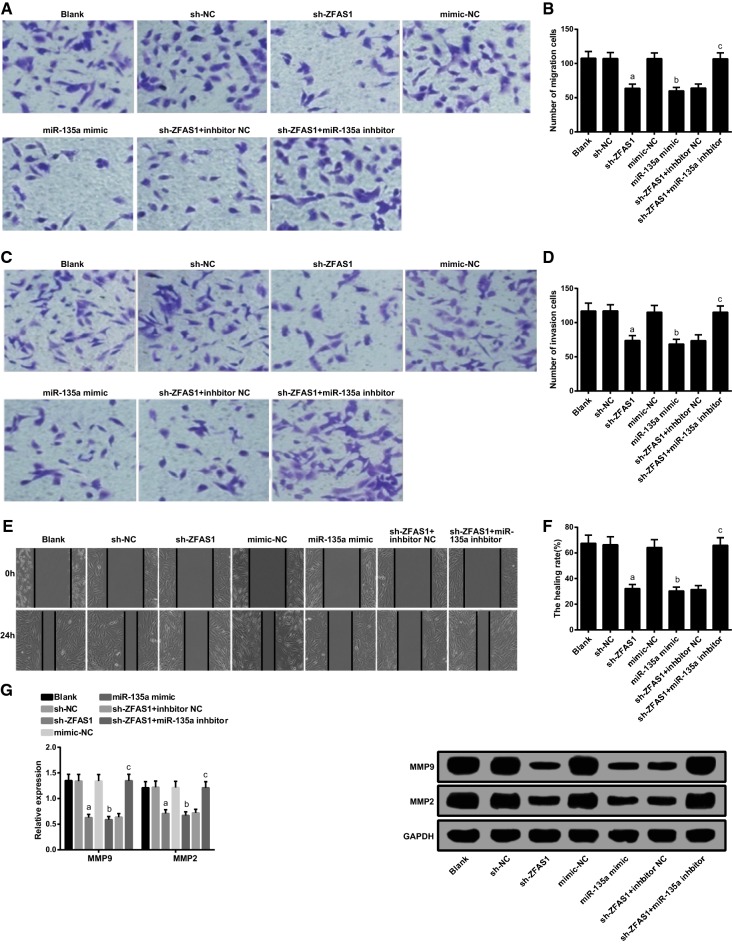
Declined ZFAS1 or up-regulated miR-135a represses MG63 cell migration and invasion. **A** MG63 cell migration tested by Transwell assay. **B** Quantification results in **A**. **C** MG63 cell invasion tested by Transwell assay. **D** Quantification results in **C**. **E** MG63 cell healing rate detected by scratch test. **F** Quantification results in **E**. **G** MMP2 and MMP9 protein bands and expression in each group of MG63 cells. The data in the figure were all measurement data, in the form of mean ± standard deviation; a vs the sh-NC group, *P* < 0.05; b vs the mimic-NC group, *P* < 0.05; c vs the sh-ZFAS1 + inhibitor NC group, *P* < 0.05

MG63 cell migration, invasion, and MMP2 and MMP9 protein expression were tested via Transwell assay, scratch test and western blot analysis, severally. Compared with the blank group, there was no distinct changes in the amount of migrated and invasive cells, the scratch healing rate, as well as MMP2 and MMP9 protein expression of MG63 cells in the sh-NC and the mimic-NC groups (all *P* > 0.05). There were decreased amount of migrated and invasive cells, scratch healing rate, MMP2 and MMP9 protein expression in the sh-ZFAS1 and the miR-135a mimic groups by contrast with the sh-NC and the mimic NC groups severally (all *P* < 0.05). In comparison to the sh-ZFAS1 + inhibitor NC group, there were elevated number of migrated and invasive cells, scratch healing rate, MMP2 and MMP9 protein expression in the sh-ZFAS1 + miR-135a inhibitor group (all *P* < 0.05) (Fig. 5A–G).

### Silenced ZFAS1 increases miR-135a in MG63 cells

**Fig. 6:**
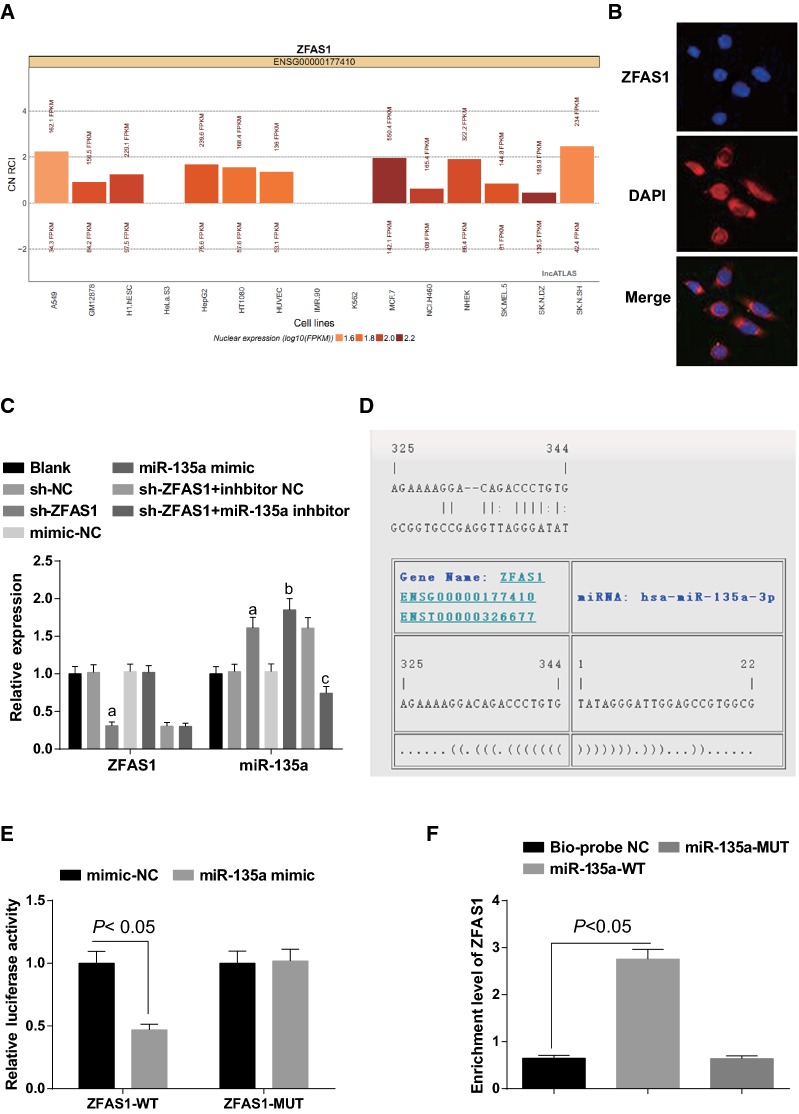
Silenced ZFAS1 increases miR-135a in MG63 cells. **A** ZFAS1 subcellular localization predicted by online analysis. **B** ZFAS1 subcellular localization verified via FISH assay. **C** ZFAS1 and miR-135a expression in each group of MG63 cells tested by RT-qPCR. **D** ZFAS1 and miR-135a binding site predicted by bioinformatics website. **E** The regulatory relationship between ZFAS1 and miR-135a verified by dual luciferase reporter gene assay. **F** The binding relationship between ZFAS1 and miR-135a verified via RNA-pull down assay. The data in the figure were all measurement data, in the form of mean ± standard deviation; a vs the sh-NC group, *P* < 0.05; b vs the mimic-NC group, *P* < 0.05; c vs the sh-ZFAS1 + inhibitor NC group, *P* < 0.05

To explore the mechanism of function of ZFAS1, through the online analysis website (http://lncatlas.crg.eu/) analysis, we found that ZFAS1 was mainly distributed in the cytoplasm (Fig. 6A). The results were verified by RNA-FISH assay and the results showed that ZFAS1 was indeed concentrated in the cytoplasm (Fig. 6B), indicating that ZFAS1 might function in the cytoplasm.

ZFAS1 and miR-135a expression in MG63 cells were detected by RT-qPCR. With the blank group by contrast, there were no obvious changes of ZFAS1 and miR-135a expression of MG63 cells between the sh-NC and the mimic-NC groups (both *P* > 0.05). By contrast with the sh-NC group, there was declined ZFAS1 and elevated miR-135a in the sh-ZFAS1 group (both *P* < 0.05). In contrast with the mimic-NC group, there was no obvious change of ZFAS1 expression in the miR-135a mimic group (*P* > 0.05), while expression of miR-135a elevated (*P* < 0.05). Versus the sh-ZFAS1 + inhibitor NC group, there were no obvious change of ZFAS1 expression in the sh-ZFAS1 + miR-135a inhibitor group (*P* > 0.05), while expression of miR-135a declined (*P* < 0.05) (Fig. 6C).

The online analysis software predicted that there was a specific binding region between the ZFAS1 gene sequence and the miR-135a sequence (Fig. 6D). The finding was further affirmed by the dual luciferase reporter gene assay that relative to the mimic-NC group, there was apparently decreased luciferase activity of WT-ZFAS1 in the miR-135a mimic group (*P* < 0.05), while there was no obvious difference of luciferase activity in the MUT-ZFAS1 (*P* > 0.05), indicating that there was a specific binding relationship between miR-135a and ZFAS1 (Fig. 6E). The results of RNA-pull down assay showed that there was higher ZFAS1 enrichment in the Bio-miR-135a-WT group in contrast to the Bio-probe NC group (*P* < 0.05), while the ZFAS1 enrichment in the Bio-miR-135a-MUT group had no obvious difference (Fig. 6F).

### Silencing ZFAS1 or up-regulated miR-135a depresses APEX1 in MG63 cells

**Fig. 7:**
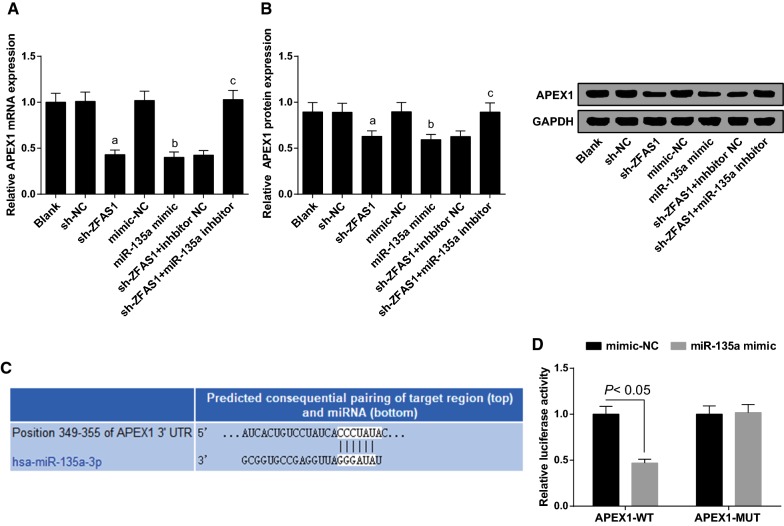
Declined ZFAS1 or elevated miR-135a represses APEX1 in MG63 cells. **A** mRNA expression of APEX1 in each group of MG63 cells detected by RT-qPCR. **B** APEX1 protein band and expression in each group of MG63 cells. **C** The binding sites of miR-135a and APEX1 predicted by bioinformatics website. **D** The regulatory relationship between miR-135a and APEX1 verified by dual luciferase reporter gene assay. The data in the figure were all measurement data, in the form of mean ± standard deviation; a vs the sh-NC group, *P* < 0.05; b vs the mimic-NC group, *P* < 0.05; c vs the sh-ZFAS1 + inhibitor NC group, *P* < 0.05

APEX1 expression in MG63 cells were detected by RT-qPCR and Western blot analysis. The results showed that compared with the blank group, there was no apparent change of APEX1 expression of MG63 cells in the sh-NC and the mimic-NC groups (*P* > 0.05). There was declined APEX1 expression in the sh-ZFAS1 and the miR-135a mimic groups by contrast with the sh-NC and the mimic NC groups severally (both *P* < 0.05). In comparison to the sh-ZFAS1 + inhibitor NC group, there was elevated APEX1 in the sh-ZFAS1 + miR-135a inhibitor group (*P* < 0.05) (Fig. 7A, B).

The target relationship between miR-135a and APEX1 was predicted by bioinformatics software http://www.targetscan.org (Fig. 7C). Results of luciferase activity assay conveyed that the relative luciferase activity of MG63 cells co-transfected with APEX1-MUT and miR-135a-mimic overtly decreased (*P* < 0.05). After MG63 cells co-transfected with APEX1-MUT and miR-135a-mimic, the relative luciferase activity of cells was not affected (*P* < 0.05) (Fig. 7D). Above results conveyed that APEX1 had a direct target relationship with miR-135a.

### Silencing ZFAS1 or elevated miR-135a decreases tumor volume and weight of OS

**Fig. 8:**
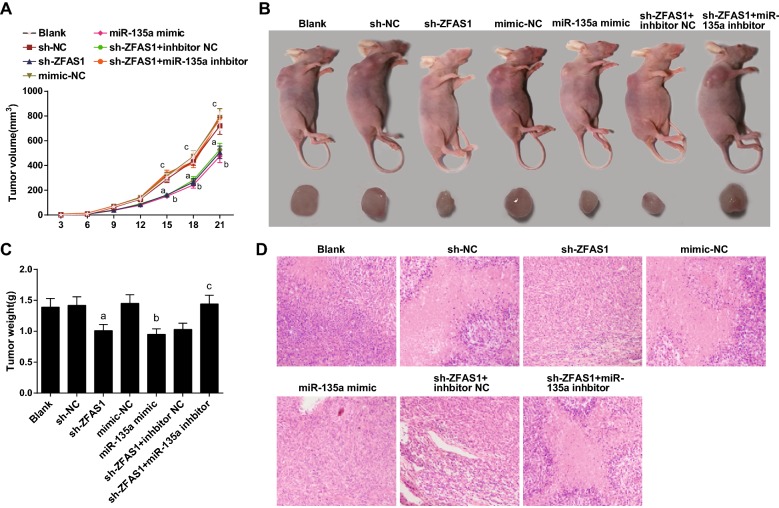
Silenced ZFAS1 or elevated miR-135a decreases tumor volume and weight of OS. **A** Tumor growth curve of nude mice in each group. **B** Entity and tumor graph on the 21st day after tumor xenograft in each group. **C** Tumor weight detection in nude mice in each group. **D** HE staining observation in each group. The data in the figure were all measurement data, in the form of mean ± standard deviation; a vs the sh-NC group, *P* < 0.05; b vs the mimic-NC group, *P* < 0.05; c vs the sh-ZFAS1 + inhibitor NC group, *P* < 0.05

The length and width of the subcutaneous tumor in nude mice were measured every 3 days, and the tumor volume was calculated based on the measured values, and the tumor growth curve was drawn. It could be seen that after 6 days of tumor xenograft, OS of each group of nude mice had varying degrees of growth. After 15 days of tumor xenograft, compared with the blank group, there was no apparent change in OS tumor volume in the sh-NC and the mimic NC groups (*P* > 0.05). There was declined OS tumor volume in the sh-ZFAS1 and the miR-135a mimic groups by contrast with the sh-NC and the mimic-NC groups severally (both *P* < 0.05). With the sh-ZFAS1 + inhibitor NC group in contrast, there were increased OS tumor volume in the sh-ZFAS1 + miR-135a inhibitor group (*P* < 0.05) (Fig. 8A).

On the 21st day after tumor xenograft, subcutaneous tumors of nude mice were seen to be more obvious. The nude mice were euthanatized, and subcutaneous OS was removed (Fig. 8B). Relative to the blank group, there was no apparent change of tumor weight in the sh-NC and the mimic-NC groups (*P* > 0.05). There was declined tumor weight in the sh-ZFAS1 and the miR-135a mimic groups by contrast with the sh-NC and the mimic NC groups severally (both *P* < 0.05). Compared with the sh-ZFAS1 + inhibitor NC group, there was elevated tumor weight in the sh-ZFAS1 + miR-135a inhibitor group (*P* < 0.05) (Fig. 8C).

The OS growing in the back of nude mice was sectioned and stained with HE solution. The section was observed under a microscope. The matrix components in OS of nude mice were overtly elevated in the blank, the sh-NC, the mimic-NC, the sh-ZFAS1 + miR-135a inhibitor groups, with decreasing cell density. There was higher cell density, less matrix components in OS in the sh-ZFAS1, the miR-135a mimic, and the sh-ZFAS1 + inhibitor NC group (Fig. 8D).

## Discussion

OS is the most general malignant bone tumor, while an urgent need is required to identify novel effective therapeutic agents for the protection of OS [14]. Evidence has demonstrated that some certain lncRNAs, miRNAs or some certain genes could be involved in the OS growth. A study has indicated that ZFAS1 can be regarded as a possible biomarker for the diagnosis and therapy of OS [8]. miR-181c was reported to be related to high-grade OS recurrence, which strengthens chemotherapeutic-induced cell death and decreases cell viability [4]. Another study has conveyed that APEX1 may be regarded as a prognostic marker and possible therapeutic target for OS [13]. This study was performed for the research of the involvement of lncRNA ZFAS1/miR-135a/APEX1 axis in the regulation of OS growth and metastasis.

In this work, we have presented evidence implicating that up-regulation of ZFAS1 existed in OS tissues. It fits well with the previously defined role that ZFAS1 expression was apparently elevated in OS tissues by contrast with corresponding noncancerous tissues [7]. There is strong evidence that ZFAS1 expression was obviously up-regulated in OS patients and for prediction of the poor prognosis, highlighting the risk factor of ZFAS1 in OS genesis [15]. A major new finding of this study was that overexpressed APEX1 was presented in OS tissues. This is consonant with the fact that increasing levels of APEX1 expression was connected with apparently declining median disease-free survival of OS patients [13]. One interesting finding was that there was down-regulated miR-135a in OS tissues. A research has found that miR-135a is declined in the most of human gastric cancer [16]. This study supports evidence from previous observations that miR-135a, a glial cell abundant of miRNA, is famous to be declined in glioma [17].

The present findings suggested that silenced ZFAS1 inhibited colony formation and proliferation, migration and invasion of MG63 cells and tumor volume and weight of OS, and promoted cell apoptosis. To further explain this mechanism, the finding in Liu et al.’ article demonstrated that ZFAS1 overexpression obviously strengthened the colony formation, migration, and invasion ability of OS cells via activating the MAPK signaling pathway [8]. Also, another convincing study has revealed that overexpressed ZFAS1 apparently facilitated cell proliferation and colony formation ability, together with migration and invasion, while silenced ZFAS1 inhibited cell migration and invasion in MG-63 cells [7]. Meanwhile, our results also suggested that up-regulated miR-135a inhibited colony formation and proliferation, migration and invasion of MG63 cells and tumor volume and weight of OS, and promoted cell apoptosis. Comparison of the findings with those of other studies confirms that overexpressing miR-135a in BGC-823 cells decreased the migratory capacity, while silencing endogenous miR-135a in SGC-7901 cells increased the migratory capacity in gastric cancer cells, indicating that miR-135a inhibits gastric cancer cell migration in vitro [18]. The other study has demonstrated that overexpression of miR-135a declined both the proliferation and colony formation ability of breast cancer cells in vitro [19].

The current study also found that silencing ZFAS1 increased miR-135a expression. A study has revealed that ZFAS1 silencing promoted miR-150 expression by direct interaction [20]. Another study has suggested that miR-150-5p had a downstream target relationship with ZFAS1 in the regulation of melanoma malignancy [6]. Except that, Yang et al. have demonstrated that miR‐432-5p expression was elevated after transfection with siRNA-ZFAS1 [21]. A finding has demonstrated that lncRNA GACAT3 performs as a molecular sponge for miR‐135a in glioma [22]. A research has also suggested that lncRNA SNHG16 can be regarded as a miR-135a sponge and restrained the function of miR-135a in the JAK2/STAT3 pathway [23]. All these evidence proves the interactions among ZFAS1 and miR-135a.

## Conclusion

In summary, this study highlights that silencing ZFAS1 or enhanced miR-135a represses proliferation, migration and invasion and promotes apoptosis of MG63 cells. This study provides a new way to further explore the pathogenesis of OS. However, more work needs to be carried out to further verify the hypothesis. Furthermore, in vivo experiments using the intra-bone injection should be conducted to verify our results.

## Data Availability

Not applicable.

## Abbreviations

OS: osteosarcoma
miR-135a: microRNA-135a
APEX1: apurinic/apyrimidinic exonuclease 1
lncRNA ZFAS1: long noncoding RNA Zinc finger antisense 1
WHO: World Health Organization
DMEM: Dulbecco’s Modified Eagle Medium
FBS: fetal bovine serum
RT-qPCR: reverse transcription quantitative polymerase chain reaction
NC: sh-negative control
OD: optical density
EdU: 5-ethynyl-2′-deoxyuridine
PBS: phosphate buffer saline
PI: propidium iodide
GAPDH: glyceraldehyde phosphate dehydrogenase
FISH: fluorescence in situ hybridization
PBST: Phosphate Buffered Saline plus Tween-20
UTR: 3′untranslated regions
WT: wild type
MUT: mutant type
ANOVA: one-way analysis of variance
